# Pulmonary nodules and CT screening: the past, present and future

**DOI:** 10.1136/thoraxjnl-2015-208107

**Published:** 2016-02-26

**Authors:** M Ruparel, S L Quaife, N Navani, J Wardle, S M Janes, D R Baldwin

**Affiliations:** 1 Lungs for Living Research Centre, UCL Respiratory, University College London, London, UK; 2 Health Behaviour Research Centre, University College London, London, UK; 3 Department of Thoracic Medicine, University College London Hospital, London, UK; 4 Respiratory Medicine Unit, David Evans Research Centre, Nottingham University Hospitals, Nottingham, UK

**Keywords:** Lung Cancer, Imaging/CT MRI etc

## Abstract

Lung cancer screening has come a long way since the early studies with chest X-ray. Advancing technology and progress in the processing of images have enabled low dose CT to be tried and tested, and evidence suggests its use can result in a significant mortality benefit. There are several issues that need refining in order to successfully implement screening in the UK and elsewhere. Some countries have started patchy implementation of screening and there is increased recognition that the appropriate management of pulmonary nodules is crucial to optimise benefits of early detection, while reducing harm caused by inappropriate medical intervention. This review summarises and differentiates the many recent guidelines on pulmonary nodule management, discusses screening activity in other countries and exposes the present barriers to implementation in the UK.

## Introduction

Individuals diagnosed with lung cancer generally have a poor prognosis, largely attributable to delayed diagnosis due to the absence of discriminating symptoms at the early stages of the disease. In the UK, it was recently estimated for the period 2010–2012 that the net 5-year survival rate of lung cancer patients was 12.7%^1^—a reflection on the 67.6% of lung cancers that are diagnosed at stage III or IV.^2^ One-year survival ranges from 14% for patients who present with stage IV disease to 71% for patients with stage I disease.^2^ Early detection has the potential to transform lung cancer outcomes and the case for screening with low radiation dose CT (LDCT) has recently gained significant momentum. However, there is a need to ensure cost-effectiveness and minimisation of harms are considered in the face of a considerable healthcare burden that has arisen from these LDCT studies; namely the management of the pulmonary nodule. In this review, we briefly discuss the pedestrian history of lung cancer screening through to the rapid evolution of the present, and highlight potential important differences in the management of the pulmonary nodule between recent guidelines.

## The history of lung cancer screening

Studies in screening for asymptomatic lung cancer began in the 1950s using photofluorograms. As early as 1959, it was clear that the lung cancer detection rate varied depending on whether medical risk was used to select the population to be screened.^3^

### Early studies using chest X-ray

Unfortunately, the trials for chest X-ray (CXR) screening for lung cancer failed spectacularly. Four large randomised controlled trials in the 1970s–1980s^4–7^ failed to detect a significant mortality benefit from CXR screening. Sadly, none of these studies used a true, null screening control group and rather compared screening with different modalities or at different frequencies. The Mayo clinic and Czechoslovakian studies compared CXR and sputum cytology at lower and higher frequencies.^4^ ^7^ Both the John Hopkins and Memorial Sloan Kettering projects compared CXR with or without sputum cytology.^5^ ^6^ Likely due to the trial designs and the short period of follow-up, no statistically significant differences in lung cancer-specific mortality were detected. However, an increase in early detection and a threefold increase in long-term survival were reported in both arms of the Memorial and Hopkins studies, when compared with the National Cancer Institutes’ Surveillance Epidemiology and End Results for unscreened cancers data.^8^ At the time, this was attributed to *lead time bias* (ie, the apparent increase in survival observed due to ‘preponing’ the diagnosis rather than the prolonging of life). Of particular note however, was an unequivocal difference in 5-year survival of early-stage detected cancers between those who had surgical resection (70%) and those who did not (10%) due to either refusal or medical contraindication,^9^ suggesting a successful early diagnosis strategy should save lives.

Despite the overall negativity following these early trials, more robust studies were carried out including the Prostate, Lung, Colorectal and Ovarian (PLCO) study which started in 1993. 155 000 smokers and non-smokers aged 55–74 were randomised to have either annual CXR for 4 years or no screening. Disappointingly, the authors reported no effect on lung cancer diagnosis, stage, histology or mortality after 13 years of follow-up.^10^ A subanalysis of the efficacy of yearly CXR screening in those at high risk also demonstrated no effect on lung cancer incidence or mortality.

### Computed tomography

The Mayo Lung Project, a North American single-arm, LDCT screening pilot carried out in 1999, detected pulmonary nodules in 74% and lung cancer in 4% of those screened. The authors concluded that LDCT could detect early-stage lung cancers but had no significant effect on mortality when compared with subjects screened by CXR in the earlier Mayo Clinic Study. They suggested that LDCT screening had led to overdiagnosis of indolent early-stage cancers and that due to the high false positive rate, the risk of complications and expense incurred in the work-up of false positive lesions, the evidence to support LDCT screening was inconclusive.^11^ ^12^

Therefore, the concept of LDCT screening for lung cancer was largely rejected until the Early Lung Cancer Action Project.^13^ ^14^ This study increased the threshold for nodule positivity to a diameter of 5mm and consequently only 13% of participants had baseline scans positive for pulmonary nodules. The prevalence lung cancer detection rate was 1.2% of all those screened and 9.7% of positive baseline scans. The majority (85%) of detected lung cancers were stage I, and these patients had an estimated 10-year survival rate of 88%. Only 8% of biopsies revealed benign lesions. These findings dramatically transformed prospects for lung cancer screening and it became apparent that deriving benefit may be possible, but further evidence from well-powered randomised studies was required. Furthermore, this study emphasised the importance of optimising protocols to manage positive screens.

This leads us to the pivotal North American, National Lung Screening Trial (NLST). This was the first well-powered randomised study that compared LDCT screening with CXR in smokers and former smokers aged 55–74. A 20% and 6.7% relative reduction in lung cancer-specific and all-cause mortality, respectively, was observed across the two groups; with a number needed to screen of 320 to save one life from lung cancer after three annual screens and seven years of follow-up.^15^ The use of CXR as a control has provoked controversy, as some argue the lack of mortality benefit observed by screening smokers and former smokers in the PLCO trial justifies CXR as equivalent to null screening, while others have argued otherwise.^16^ Another limitation of this study is that the majority of NLST participants were younger, white, well-educated and affluent, while higher risk individuals were under-represented. This brings us to two important observations. First, the failure to engage those most at risk of lung cancer in screening in the NLST study may have led to an underestimation of the potential benefit of screening. But second, this study importantly highlights the difficulties faced in undertaking a cost-effective screening approach across society.

NLST radically changed prospects for LDCT screening, but can these data be extrapolated to the UK and Europe? One of the aims of the UK Lung Cancer Screening Trial (UKLS) was to address this and to evaluate costs within the UK National Health Service (NHS).^17^ Several other trials in Europe have also recruited, but those that reported on mortality were substantially underpowered and failed to detect a benefit.^18–23^ Combining the populations within these studies together with the Dutch-Belgian lung cancer screening trial (NELSON), the Danish Lung Cancer Screening Trial and UKLS will amount to a total of approximately 36 000 participants; although, with varying nodule management algorithms and criteria for eligibility. The pooled results are eagerly awaited by physicians and health providers alike, and should mature in 2016.^24^

## LDCT: management of pulmonary nodules

The studies discussed have clarified the CT features of nodules and growth rates that support benign or malignant diagnoses.^25–30^ Several predictive models taking into account clinical and demographic factors, as well as CT and positron emission tomography (PET) features of nodules have been proposed and validated, enabling quantification of risk of malignancy for a given nodule.^31–33^ As a result, we can adopt a more conservative approach to certain nodules by employing CT surveillance, reserving the more invasive procedures for higher risk nodules.

In 2005, in response to the growing problem of small CT-detected nodules, the Fleischner Society published a management algorithm.^34^ The strategy adopted was a conservative one that mandated that all small nodules should be followed-up in high-risk people (essentially smokers or former smokers). More recent guidelines, published by the American College of Chest Physicians (ACCP),^35^ generally mirror the Fleischner guidelines with little change to follow-up recommendations (table 1). The Fleischner Society have recently responded to the problem of the subsolid nodule by publishing a further statement on their management.^36^ For those who are at high risk as per the US Preventative Services Task Force (USPSTF) criteria, the Lung CT Screening Reporting and Data System (Lung-RADS) has been specifically created.^37^ However, there is emerging evidence that implementation of the ACCP guidelines in the US has been suboptimal and that performance of Lung-RADS may not be as accurate as an approach using the Brock University nodule risk prediction model.^38^ Tables 1–3 show the comparison between these various nodule management strategies, which vary considerably.

**Table 1:** Comparison of Fleischner, Lung-RADS and ACCP guideline management of SN detected within or outside of screening

	Fleischner		ACCP		Lung-RADS
	Low risk	High risk	Low risk	High risk	
Baseline scan					
No nodules or nodules with benign features	No follow-up	No follow-up	Not specified		Category 1 (negative): return to annual screening at 12 months
<4 mm		Interval CT at 12 months	Optional follow-up	Interval CT at 12 months then discharge if stable	Category 2 (benign): return to annual screening at 12 months
4–6 mm	Interval CT at 12 months	Interval CT at 6–12 and 18–24 months	Interval CT at 12 months then discharge if stable	Interval CT at 6–12 and 18–24 months and discharge at 24 months if stable	
6–8 mm	Interval CT at 6–12 and 18–24 months	3–6, 9–12 and 24 months	Interval CT at 6–12 and 18–24 months and discharge at 24 months if stable	Interval CT at 3–6, 9–12 and 24 months and discharge at 24 months if stable	Category 3 (probably benign): interval CT at 6 months
8–15 mm	Interval CT at 3, 9, 24 months, dynamic CT chest, PET-CT±histology		Risk<5%: perform CT surveillance at 3–6, 9–12 and 18–24 months If non-FDG avid/ non-enhancing on contrast CT and risk <40% or negative biopsy: perform CT surveillance at 3–6, 9–12 and 18–24 months Risk 5%–65%: perform PET and consider biopsy/resection Risk >65%: PET not required favour histological confirmation, with surgical resection		Category 4A (suspicious): PET-CT: interval CT at 3 months
>15 mm					Category 4B (suspicious): perform standard CT with or without contrast; PET-CT; histology
Interval scan					
<4 mm (new)	Discharge if resolved or at 12 months if no growth		If there is clear evidence of growth (VDT<400 days is suggestive of malignancy), favour surgical resection If no growth or decrease in size, follow-up for 2 years or until nodule disappears		Category 2 (benign): return to annual screening at 12 months
4–6 mm (new)	Discharge if resolved or at 1 year if no growth	Discharge if resolved or at 18–24 months if no growth			Category 3 (probably benign): interval CT at 6 months
<8 mm (new/growing)	Discharge if resolved or at 24 months if no growth	Discharge if resolved or at 24 months if no growth			Category 4A (suspicious): interval CT at 3 months
>8 mm (new/growing)	Perform PET-CT±histology Discharge if resolved or at 24 months if no growth				Category 4B (suspicious): perform standard CT with or without contrast; PET-CT; histology

N.B. Lung-RADS is for CT screening scans only.

ACCP, American College of Chest Physicians; PET, positron emission tomography; RADS, Reporting and Data System; SN, solid nodules.

**Table 2:** Comparison of Fleischner, Lung-RADS and ACCP guideline management of PSN detected at baseline screening or incidental scans

	Fleischner	ACCP	Lung-RADS
Baseline scan			
No nodules or nodules with benign features	Interval CT at 3 months	If <8 mm: interval CT at 3 and 12 months and then annually for 3–5 years If multiple, consider each nodule separately	Category 1 (negative): interval CT at 12 months
<6 mm			Category 2 (benign): interval CT at 12 months
≥6 mm with solid component <6 mm			Category 3 (probably benign): interval CT at 6 months
≥6 mm with solid component 6–8 mm			Category 4A (suspicious): interval CT at 3 months; PET-CT if solid component >8 mm
≥6 mm with solid component ≥8 mm		Interval CT at 3 months+PET if solid component >8 mm N.B. If >15 mm, consider PET+biopsy/resection at baseline	Category 4B (suspicious): standard CT with or without contrast; PET-CT if solid component ≥8 mm; histology
Interval scan			
<6 mm nodule	Persistent nodules with solid component <5 mm: perform annual CT for minimum 3 years	Annual CT for 3–5 years. Any growth or development of solid component should prompt further investigation/resection	<6 mm nodule: category 2 (benign): interval CT at 12 months
≥6 mm with smaller solid component			≥6 mm nodule solid component <6 mm (or new nodule <6 mm on interval CT): category 3 (probably benign): interval CT at 6 months
≥6 mm with larger solid component	For persistent solitary nodule or multiple nodules with one dominant nodule with solid component >5 mm: favour biopsy/resection (PET-CT if nodule >10 mm)	Perform PET-CT+biopsy/resection	≥6 mm nodule with solid component 6–8 mm (or new or growing solid component <4 mm): category 4A (suspicious): interval CT at 3 months
			≥6 mm with solid component >8 mm or new or growing solid component >4 mm: category 4B (suspicious): standard CT with or without contrast; PET-CT; histology depending on Brock risk

N.B. Lung-RADS is for CT screening scans only.

ACCP, American College of Chest Physicians; PET, positron emission tomography; PSN, part-solid nodules; RADS, Reporting and Data System.

**Table 3:** Comparison of Fleischner, Lung-RADS and ACCP guideline management of pGGN detected at baseline screening or incidental scans

	Fleischner	ACCP	Lung-RADS
Baseline scan			
<5 mm	No follow-up if solitary, but if multiple, perform interval CT at 2 and 4 years	No follow-up	See below
>5 mm	Interval CT at 3 months, then annual for minimum 3 years (for solitary and multiple nodules)	Annual CT surveillance for minimum 3 years (follow-up at 3 months if >10 mm)	
<20 mm	As above		Category 2 (benign): interval CT at 12 months
≥20 mm			Category 3 (probably benign): interval CT at 6 months
Interval scan			
New nodule			
<20 mm	As for baseline scan	If >10 mm and persistent or growing favour resection	Category 2 (benign): interval CT at 12 months
≥20 mm and stable or slow growth			Category 3 (probably benign): interval CT at 6 months
Persistent nodule	Annual CT surveillance for a minimum of 3 years Favour excision if nodule >10 mm or multiple pGGN with a persistent dominant nodule Biopsy noted to have low yield		>20 mm and stable or slowly growing=category 2 (benign): interval CT at 12 months

N.B. Lung-RADS is for CT screening scans only.

ACCP, American College of Chest Physicians; pGGN, pure ground glass nodules; RADS, Reporting and Data System.

The British Thoracic Society (BTS) published new guidelines on the investigation and management of pulmonary nodules in July 2015 following a comprehensive review of the evidence, with a third of the references cited from 2012 or later.^39^ Importantly, recommendations differ substantially from the earlier guidelines, especially for very small nodules, and recommend higher nodule follow-up thresholds, the use of risk prediction calculators and automated volumetric assessment to clarify follow-up requirements and growth rates (table 4). The inclusion of volumetric measurement will be challenging to implement across the UK but this will be offset by a substantial reduction in follow-up scans compared with previous guidelines. The Brock University risk prediction tool, which was developed from the Pan-Canadian screening cohort^32^ and the Herder model, where PET-CT results are available, are recommended to more accurately define risk of malignancy. The role of further imaging, minimally invasive investigations and therapy is reviewed and recommendations made. This guideline also includes a service delivery model.

**Table 4:** Summary of BTS guidelines for management of pulmonary nodules detected at baseline screening or incidental scans

	Solid nodules	Part solid nodules	Pure ground glass nodules
Baseline scan			
<5 mm or <80 mm^3^	Discharge	Discharge	
5–6 mm	CT at 12 months	Interval CT at 3 months	
≥6 to <8 mm or 80 to <300 mm^3^	Interval CT at 3 and 12 months		
>8 mm or >300 mm^3^	PET-CT Assess Herder risk ▸ If <10%: do CT at 3 and 12 months ▸ If >10%: consider biopsy or resection or CT surveillance on individual basis ▸ If >70% favour resection		
Interval scan			
2D		If stable or smaller, assess Brock risk at 3 months ▸ If risk <10%, continue CT surveillance at 1, 2 and 4 years (or until nodule disappears) ▸ If risk >10% or concerning morphology, consider histological diagnosis and discuss options with patient ▸ If growth or altered morphology (especially if growth of solid component by ≥2 mm) favour further work up and definitive management N.B. Consider PET if solid component ≥8 mm	
Stable nodule	Discharge after 2 years		
Growing	PET-CT and Herder score		
3D			
Stable or slow growth	Discharge if stable, and discharge or ongoing surveillance if slow growth (VDT>600 days)		
VDT 400–600 days	Further surveillance or biopsy or resection are acceptable, and decision should be based on patient preference		
VDT ≤400 days	Further work up, and consider definitive management		

BTS, British Thoracic Society; PET, positron emission tomography; 2D, two-dimensional; 3D, three-dimensional.

## Current CT screening activity inside and outside the UK

Lung cancer screening by CXR was advocated by the American Cancer Society in the 1970s, however this recommendation was withdrawn following evidence from the trials in the 1980s. Following the publication of the NLST results, the USPSTF recommended screening with LDCT of individuals aged 55–80 who have accrued at least a 30 pack-year smoking history and are current or former smokers who have given up for ≤15 years. The ACCP/American Thoracic Society, American College of Radiology (ACR) and National Comprehensive Cancer Network have all also released statements or guidelines for screening.^40–42^

In February 2015, the US insurers, the Center for Medicare and Medicaid Services agreed to fund screening of asymptomatic insured individuals aged 55–77 who meet the USPSTF smoking criteria.^43^ The newly instated ‘Obamacare’ enables some 30% of the uninsured population to access LDCT screening; however, once a nodule is detected it is classified as surveillance rather than screening, and perversely funds are insufficient to cover this crucial aspect of the screening process. Furthermore, a significant proportion of the US population do not qualify for screening at all through lack of insurance coverage. What is really required, therefore, is a national screening programme that is accessible to individuals from all communities such as the US National Breast and Cervical Cancer Early Detection Program.^44^ Nonetheless, LDCT screening for insured individuals is now underway in the US. The ACR runs an accreditation programme outlining basic standards for performing and evaluating screening scans using the above-mentioned Lung-RADS.^42^ At the time of writing, there were 1220 accredited centres for LDCT screening in the US, 43 of which had been awarded ‘diagnostic imaging centre of excellence’. The ACR has constructed a lung cancer screening registry, to record outcomes from screening and the first feedback was expected in autumn 2015.^45^

Screening has not yet been initiated in Canada, and the European Society of Radiology and European Respiratory Society recommend that screening should be performed in ‘comprehensive, quality-controlled longitudinal programmes’.^46^ In China, several lung cancer screening programmes have been initiated, particularly in areas with high lung cancer incidence, funded by central or local government.^47^

The UK National Screening Committee (NSC) is due to make a decision on lung cancer LDCT screening in the UK pending the results of the pooled European data.^48^ In the meantime, several centres around the UK have initiated early diagnosis campaigns or pilot screening projects and these will contribute increasing knowledge around the best methods of implementation in the UK.^49^

## Present barriers to successful implementation of LDCT screening

With these advances in CT technology and pulmonary nodule management, are we now in a position to recommend screening? In 1968, Wilson and Jungner compiled a report commissioned by the WHO highlighting that while the concept of screening was admirable, it was not without difficulties in terms of optimising benefits and harms.^50^ In order to aid the appropriate selection of conditions for which the benefits of screening outweighed the harms, they outlined 10 screening principles for appraising the viability, effectiveness and appropriateness of a screening programme,^51^ which form the basis for the criteria outlined by the UK NSC. Table 5 lists these 10 principles, and proposes key factors that need to be addressed for successful implementation of a lung cancer screening programme. These factors are discussed below.

**Table 5:** Current issues to be addressed for implementation to lung cancer LDCT screening in the UK with reference to Wilson and Jungner^50^ criteria

WHO Wilson and Jungner^50^ criteria	Met	Key factors for implementation
1. The condition sought should be an important health problem	Yes	
2. There should be an accepted treatment for patients with recognised disease	Yes	
3. Facilities for diagnosis and treatment should be available		Resource implications and availability of volumetric assessment
4. There should be a recognisable latent or early symptomatic stage	Yes	Overdiagnosis and false positive rates
5. There should be a suitable test or examination	Yes	Radiation risk
6. The test should be acceptable to the population	Yes	Balance of psychological impact
		Equitable access, uptake and adherence to screening across the population
7. The natural history of the condition, including development from latent to declared disease, should be adequately understood	Yes	
8. There should be an agreed policy on whom to treat as patients		Optimal eligibility criteria for screening
9. The cost of case finding (including diagnosis and treatment of patients diagnosed) should be economically balanced in relation to possible expenditure on medical care as a whole	Yes	Co-implementation of smoking cessation
		Incidental findings from screening
10. Case-finding should be a continuing process and not a ‘once and for all’ project		Interval cancer rate
		Regulating CT screening

LDCT, low dose CT.

### Radiation risk

Advances in CT scanning technology have improved nodule detection and characterisation while reducing radiation dose. Techniques such as iterative reconstruction (which reduces the extent of noise and artefact associated with images at lower radiation doses), altering tube voltage, current and gantry rotation speed are valuable in reducing effective radiation doses. Further reduction to overall radiation exposure over time will follow implementation of the more conservative approaches to managing positive findings, suggested in the more recent guidelines.^52^ Nevertheless, radiation risk will remain a problem and always needs to be considered when balancing risks and benefits.

### Overdiagnosis and false positives

Overdiagnosis is the detection and characterisation of disease that would not otherwise cause harm. It may be minimised in LDCT screening by taking into account morphological features that denote more indolent cancers that may be managed with a more conservative approach less likely to cause harm. False positives are benign lesions that undergo investigation and may therefore result in harm, both physical and psychological. Pure ground glass nodules^53^ and solid and subsolid nodules with longer volume doubling times are more likely to be indolent^30^ or benign. The recent BTS guidelines support a more conservative management of volumetrically assessed, potentially indolent or benign lesions, thus reducing the rate of overdiagnosis and benign histological diagnoses.

### Optimal eligibility criteria for screening

It is clear that overdiagnosis rates, false positive rates, the number needed to be screened to save one life from lung cancer and cost of screening can all be reduced by selecting the higher risk population.^54^ However, what the appropriate threshold for risk is, and how this interacts with entry and exit age, competing mortality and fitness are complex. Randomised trials of LDCT screening have adopted a number of different approaches in determining who to invite to screening. The UKLS sent out questionnaires which enabled determination of the Liverpool Lung Project lung cancer risk prediction algorithm and had a relatively high lung cancer detection rate (2.1%),^55^ while the NLST used just age and smoking status. Which risk assessment tool most appropriately balances simplicity and predictive accuracy still needs to be determined. Furthermore, based on the results from the NELSON study, screening interval may also need to be varied by risk. They showed that interval cancers occur at a low rate (1%) and this occurrence was associated with age but not smoking status.^56^ Further research into other factors predictive of interval cancers is needed. With ongoing research into potential biomarkers, LDCT screening may one day be offered to a wider demographic and include non-smokers with a positive biomarker test.

### Balance of psychological impact

The psychological burden of screening remains to be determined, and for the individual, may in large part depend on the severity of the screening result and psychosocial characteristics. To date, most studies of trial participants undergoing lung screening have shown that the anxiety and distress associated with false positive screens are minimal and short-lived.^57^ Clinically, meaningful changes in health-related quality of life were observed one month after the detection of an indeterminate pulmonary nodule, but returned to prescreening levels after two years, suggesting no long-lasting effects.^58^ ^59^ However, this is yet to be studied in the community context and there is evidence from other screening programmes for long-term psychological distress.^60^ Furthermore, given a significant proportion of screening participants will at one time receive a false positive or indeterminate result, further research is needed to monitor the psychological impact of the surveillance process, examine individual differences in response and develop strategies which minimise harm. Qualitative evidence suggests effective communication about indeterminate nodules by health professionals could be one such strategy,^61^ and ensuring patients are well-informed about the screening process and possible results will be essential. In addition, there is concern that an ‘all clear result’ could be falsely interpreted to mean low future risk of developing lung cancer, and a lower susceptibility to the effects of smoking. This issue of over-reassurance has been studied for other cancer types, with evidence that it may compromise future symptom appraisal and delay symptomatic help-seeking.^62^ Care must therefore be taken to tailor individual communication, so as to minimise adverse psychological responses to screening and any negative impact on future health behaviours.

### Incidental findings from screening

Incidental findings are often viewed as a negative aspect of CT screening, but their detection may also provide an opportunity to rectify other conditions that threaten quality of life or survival. The NLST reported a 6.7% reduction in all-cause mortality, an effect most pronounced in Black African–Americans.^15^ ^63^ This may be explained by detection of clinical and radiological findings in the process of screening, and due to the improved access to healthcare brought about by screening that may prompt intervention for non-lung cancer co-morbidities.^64^ Several studies have shown that ungated LDCT scans can accurately predict coronary calcium and subsequent cardiovascular events comparable to formal coronary calcium scoring.^65^ ^66^ Those at high risk of lung cancer are also at higher risk of cardiovascular disease, and combining risk assessment and screening provides opportunity to improve outcomes for both conditions. However, further prospective studies are needed. LDCT scans have also been shown to be useful for detecting emphysema,^67^ which is a recognised risk factor for lung cancer, and osteoporosis which was associated with all-cause mortality in the NELSON cohort.^68^

### Co-implementation of smoking cessation

Achieving smoking abstinence in combination with LDCT screening has been reported to almost double the reduction in lung cancer mortality compared with screening alone within the NLST participants,^63^ but the impact of screening on smoking cessation is unclear. Several studies have reported increased smoking cessation in trial participants compared with the background population. However, no significant differences in outcomes between the screened and control groups have been noted, suggesting that trial participants may be a more motivated group.^69–75^ Few studies have noted an increase in the number of participants abstaining from smoking with successive positive or indeterminate screen results compared with those with negative screens.^76–78^ The optimal method of promoting smoking cessation in screening participants has not been determined. Further studies of patients, rather than trial participants, in the real-life screening context are needed to further explore this.

### Resource implications and availability of volumetric assessment

Several studies, based primarily on US data, have reported varying Incremental Cost Effectiveness Ratios per quality of life adjusted life year (QALY) gained, ranging from US$28 000 to over US$100 000.^79–81^ The cost of lung cancer screening in the UK is not known, but has been estimated to be around £9000 per QALY gained;^55^ well below the threshold of £30 000 deemed acceptable by the National Institute for Health and Care Excellence. If NLST eligibility criteria were to be implemented, an estimated 8.7 million people in the US may be eligible for screening.^82^ The size of the UK lung cancer screening eligible population is unclear, however, even assuming uptake levels may be low (in the region of 50%), numbers are likely to be significant. This has considerable resource implications in terms of carrying out the baseline and interval CT scans required for nodule follow-up.

Most hospitals now have the technology to perform LDCT scans and take advantage of many of the other CT advances such as Maximum Intensity Projections (which allow enhanced visualisation of high attenuating structures such as nodules) and Multi-Planar Reconstruction (where images are reconstructed in customised planes). Automated estimation of the volume of nodules, calculated by mapping the CT attenuation values, is also possible with modern volumetric nodule assessment packages. These packages also autocalculate volume doubling times, which can more accurately quantify growth of a nodule than conventional two-dimensional measurements.^83^ However, exactly what proportion of UK hospitals currently has access to the technical, radiological and clinical know-how to implement volumetric assessment needs to be determined.

Various strategies as outlined above including appropriate selection criteria for screening and improved management of pulmonary nodules will help reduce the cost of screening. The cost of various methodologies that can be used in the screening process, such as the use of mobile CT scanners versus dedicated screening centres, also needs to be evaluated. Scan reading time is also a factor, and research is needed to explore whether there are feasible options that will relieve some of the work from radiologists.

### Equitable access, uptake and adherence to screening across the population

Participation in national screening programmes for breast and colorectal cancer is around 70% and 60%, respectively,^84–87^ but there is evidence to suggest this will be lower in future lung cancer screening programmes. In the NELSON trial, 32% of the approached persons (aged 50–75 years) in the general population responded to an initial questionnaire on general health, lifestyle and smoking history (which did not mention the NELSON trial^88^), similar to the response rate for the UKLS pilot randomised control trial.^89^ In NELSON, 19% (6% overall) of these were at high risk and half gave informed consent and were recruited. In UKLS, only 11.5% met the risk threshold for trial entry (higher than for NELSON) and a similar proportion were recruited. Although participation in a trial might be less than in a screening programme, in both of these well-conducted studies the participation rate of higher risk people was low enough to be of concern for implementation.

UKLS also showed that current smoking and low socioeconomic status (SES) were associated with lower uptake, a problem observed across European and US trials^90^ ^91^ and for other cancer screening programmes more widely.^92^ Lung cancer prevalence is higher in lower SES communities, where lifelong smokers are both over-represented^93^ and more tobacco-dependent.^94^ Indeed, over 40% of the lung cancers detected in UKLS were in people from the most deprived quintile. Reported barriers to participation among smokers include concern about risk of lung cancer, a lower perceived benefit of early detection, perceived blame and stigma and fearful, fatalistic and nihilistic beliefs around lung cancer outcomes.^95–97^ In addition, methods of recruitment, which demand significant correspondence from potential participants (as required by most trials), are likely to increase attrition among lower SES groups. A UK study has begun recruitment to a trial examining a tailored invitation method designed to overcome these barriers and reduce inequalities in participation (Quaife *et al*, manuscript in preparation).

Factors affecting screening adherence may be similar to those affecting uptake, with the added complexity of psychological responses to positive and negative results received in previous screening rounds.^98^ Given that the ratio of screening benefit to risk increases with lung cancer risk, promoting engagement of the higher risk and hard-to-reach groups aims to reduce lung cancer inequalities and improve the cost-effectiveness and efficacy of screening.

### Regulating CT screening

Any screening programme needs to have stringent audit and quality control to ensure compliance with best practice. Availability of adequate resources to successfully implement and deliver such a programme to a high standard is vital. Controls also need to be in place to ensure uniformly low radiation doses are used, scans are read to an adequate level of accuracy and that nodules and other findings are appropriately managed. A database to record outcomes is crucial to enable measurement of screening outcomes and further develop and improve the protocols used for all aspects of screening.

## Conclusion

LDCT screening is undoubtedly a promising method to improve lung cancer outcomes. If lung cancer screening is to be initiated in the UK, adequate provision of resources is essential, with employment of stringent screening protocols and regulatory processes to ensure benefits outweigh harms and costs are minimised. Although it is acknowledged that the UK NHS is under considerable resource pressure, lung cancer is a condition that has not seen the improved outcomes observed in other cancers. With an overall 5-year survival for lung cancer in the UK <13%^2^ and the limited efficacy of available treatments for late-stage disease, there seems to be no alternative but to proceed with screening to improve rates of early detection and curative treatment.

## References

[1] Walters S, Benitez-Majano S, Muller P, et al Is England closing the international gap in cancer survival? Br J Cancer 2015;113:848–60. 10.1038/bjc.2015.265 26241817 PMC4559829

[2] Cancer Stats: Key Stats 2015 http://www.cancerresearchuk.org/cancer-info/cancerstats/keyfacts/ (accessed 22 May 2015).

[3] BRETT GZ Bronchial carcinoma in men detected by selective and unselective miniature radiography: a review of 228 cases. Tubercle 1959;40:192–5. http://www.ncbi.nlm.nih.gov/pubmed/13804288 (accessed 22 Jul 2015). 10.1016/S0041-3879(59)80040-4 13804288

[4] Marcus PM, Bergstralh EJ, Fagerstrom RM, et al Lung cancer mortality in the Mayo Lung Project: impact of extended follow-up. J Natl Cancer Inst 2000;92:1308–16. http://www.ncbi.nlm.nih.gov/pubmed/10944552 (accessed 22 Jul 2015). 10.1093/jnci/92.16.1308 10944552

[5] Frost JK, Ball WC, Levin ML, et al Early lung cancer detection: results of the initial (prevalence) radiologic and cytologic screening in the Johns Hopkins study. Am Rev Respir Dis 1984;130:549–54. 10.1164/arrd.1984.130.4.549 6091505

[6] Melamed MR, Flehinger BJ, Zaman MB, et al Screening for early lung cancer. Results of the Memorial Sloan-Kettering study in New York. Chest 1984;86:44–53. 10.1378/chest.86.1.44 6734291

[7] Kubík A, Polák J Lung cancer detection. Results of a randomized prospective study in Czechoslovakia. Cancer 1986;57:2427–37. 10.1002/1097-0142(19860615)57:12<2427::AID-CNCR2820571230>3.0.CO;2-M 3697941

[8] Strauss GM, Gleason RE, Sugarbaker DJ Chest X-ray screening improves outcome in lung cancer. A reappraisal of randomized trials on lung cancer screening. Chest 1995;107:270S–9S. 10.1378/chest.107.6_Supplement.270S 7781405

[9] Flehinger BJ, Kimmel M, Melamed MR The effect of surgical treatment on survival from early lung cancer. Implications for screening. Chest 1992;101:1013–18. 10.1378/chest.101.4.1013 1313349

[10] Oken MM, Hocking WG, Kvale PA, et al Screening by chest radiograph and lung cancer mortality: the Prostate, Lung, Colorectal, and Ovarian (PLCO) randomized trial. JAMA 2011;306:1865–73. 10.1001/jama.2011.1591 22031728

[11] Swensen SJ, Jett JR, Hartman TE, et al CT screening for lung cancer: five-year prospective experience. Radiology 2005;235:259–65. 10.1148/radiol.2351041662 15695622

[12] Swensen SJ, Jett JR, Hartman TE, et al Lung cancer screening with CT: Mayo Clinic experience. Radiology 2003;226:756–61. 10.1148/radiol.2263020036 12601181

[13] Henschke CI, McCauley DI, Yankelevitz DF, et al Early Lung Cancer Action Project: overall design and findings from baseline screening. Lancet 1999;354:99–105. 10.1016/S0140-6736(99)06093-6 10408484

[14] Henschke CI, Yankelevitz DF, Libby DM, et al Survival of patients with stage I lung cancer detected on CT screening. N Engl J Med 2006;355:1763–71. 10.1056/NEJMoa060476 17065637

[15] Aberle DR, Adams AM, Berg CD, et al, National Lung Screening Trial Research Team. Reduced lung-cancer mortality with low-dose computed tomographic screening. N Engl J Med 2011;365:395–409. 10.1056/NEJMoa1102873 21714641 PMC4356534

[16] Reich JM, Kim JS The National Lung Screening Trial premise of null and chest radiography equivalence is open to question. AJR Am J Roentgenol 2015;205:278–9. 10.2214/AJR.14.13945 26204275

[17] Field JK, Hansell DM, Duffy SW, et al CT screening for lung cancer: countdown to implementation. Lancet Oncol 2013;14:e591–600. 10.1016/S1470-2045(13)70293-6 24275132

[18] Infante M, Lutman FR, Cavuto S, et al Lung cancer screening with spiral CT: baseline results of the randomized DANTE trial. Lung Cancer 2008;59:355–63. 10.1016/j.lungcan.2007.08.040 17936405

[19] Lopes Pegna A, Picozzi G, Mascalchi M, et al Design, recruitment and baseline results of the ITALUNG trial for lung cancer screening with low-dose CT. Lung Cancer 2009;64:34–40. 10.1016/j.lungcan.2008.07.003 18723240

[20] Pastorino U, Rossi M, Rosato V, et al Annual or biennial CT screening versus observation in heavy smokers: 5-year results of the MILD trial. Eur J Cancer Prev 2012;21:308–15. 10.1097/CEJ.0b013e328351e1b6 22465911

[21] Pedersen JH, Ashraf H, Dirksen A, et al The Danish randomized lung cancer CT screening trial—overall design and results of the prevalence round. J Thorac Oncol 2009;4:608–14. 10.1097/JTO.0b013e3181a0d98f 19357536

[22] Veronesi G, Maisonneuve P, Rampinelli C, et al Computed tomography screening for lung cancer: results of ten years of annual screening and validation of cosmos prediction model. Lung Cancer 2013;82:426–30. 10.1016/j.lungcan.2013.08.026 24099665

[23] Blanchon T, Bréchot JM, Grenier PA, et al Baseline results of the Depiscan study: a French randomized pilot trial of lung cancer screening comparing low dose CT scan (LDCT) and chest X-ray (CXR). Lung Cancer 2007;58:50–8. 10.1016/j.lungcan.2007.05.009 17624475

[24] Field JK, van Klaveren R, Pedersen JH, et al European randomized lung cancer screening trials: post NLST. J Surg Oncol 2013;108:280–6. 10.1002/jso.23383 23893464

[25] Harders SW, Madsen HH, Rasmussen TR, et al High resolution spiral CT for determining the malignant potential of solitary pulmonary nodules: refining and testing the test. Acta Radiol 2011;52:401–9. 10.1258/ar.2011.100377 21498302

[26] de Hoop B, van Ginneken B, Gietema H, et al Pulmonary perifissural nodules on CT scans: rapid growth is not a predictor of malignancy. Radiology 2012;265:611–16. 10.1148/radiol.12112351 22929331

[27] Henschke CI, Yankelevitz DF, Yip R, et al Lung cancers diagnosed at annual CT screening: volume doubling times. Radiology 2012;263:578–83. 10.1148/radiol.12102489 22454506 PMC3329268

[28] Ashraf H, Dirksen A, Loft A, et al Combined use of positron emission tomography and volume doubling time in lung cancer screening with low-dose CT scanning. Thorax 2011;66:315–19. 10.1136/thx.2010.136747 21169285

[29] Mehta HJ, Ravenel JG, Shaftman SR, et al The utility of nodule volume in the context of malignancy prediction for small pulmonary nodules. Chest 2014;145:464–72. 10.1378/chest.13-0708 23949741 PMC3941244

[30] Horeweg N, van Rosmalen J, Heuvelmans MA, et al Lung cancer probability in patients with CT-detected pulmonary nodules: a prespecified analysis of data from the NELSON trial of low-dose CT screening. Lancet Oncol 2014;15:1332–41. 10.1016/S1470-2045(14)70389-4 25282285

[31] Herder GJ, van Tinteren H, Golding RP, et al Clinical prediction model to characterize pulmonary nodules: validation and added value of 18F-fluorodeoxyglucose positron emission tomography. Chest 2005;128:2490–6. 10.1378/chest.128.4.2490 16236914

[32] McWilliams A, Tammemagi MC, Mayo JR, et al Probability of cancer in pulmonary nodules detected on first screening CT. N Engl J Med 2013;369:910–19. 10.1056/NEJMoa1214726 24004118 PMC3951177

[33] Al-Ameri A, Malhotra P, Thygesen H, et al Risk of malignancy in pulmonary nodules: a validation study of four prediction models. Lung Cancer 2015;89: 27–30. 10.1016/j.lungcan.2015.03.018 25864782

[34] MacMahon H, Austin JHM, Gamsu G, et al Guidelines for management of small pulmonary nodules detected on CT scans: a statement from the Fleischner Society. Radiology 2005;237:395–400. 10.1148/radiol.2372041887 16244247

[35] Gould MK, Donington J, Lynch WR, et al Evaluation of individuals with pulmonary nodules: when is it lung cancer? Diagnosis and management of lung cancer, 3rd ed: American College of Chest Physicians evidence-based clinical practice guidelines. Chest 2013;143:e93S–120S. 10.1378/chest.12-2351 23649456 PMC3749714

[36] Naidich DP, Bankier AA, MacMahon H, et al Recommendations for the management of subsolid pulmonary nodules detected at CT: a statement from the Fleischner Society. Radiology 2013;266:304–17. 10.1148/radiol.12120628 23070270

[37] American College of Radiology. Lung CT Screening Reporting and Data System (Lung-RADS™). American College of Radiology http://www.acr.org/Quality-Safety/Resources/LungRADS (accessed 20 Aug 2015).

[38] Tanner NT, Aggarwal J, Gould MK, et al Management of pulmonary nodules by community pulmonologists: a multicenter observational study. Chest 2015;148:1405–14. 10.1378/chest.15-0630 26087071 PMC4665735

[39] Baldwin DR, Callister ME The British Thoracic Society guidelines on the investigation and management of pulmonary nodules. Thorax 2015;70:794–8. 10.1136/thoraxjnl-2015-207221 26135833

[40] Mazzone P, Powell CA, Arenberg D, et al Components necessary for high quality lung cancer screening: American College of Chest Physicians and American Thoracic Society policy statement. Chest 2015;147:295–303. 10.1378/chest.14-2500 25356819 PMC4502754

[41] Wood DE National Comprehensive Cancer Network (NCCN) Clinical Practice Guidelines for Lung Cancer Screening. Thorac Surg Clin 2015;25:185–97. 10.1016/j.thorsurg.2014.12.003 25901562

[42] Kazerooni EA, Armstrong MR, Amorosa JK, et al ACR CT accreditation program and the lung cancer screening program designation. J Am Coll Radiol 2015; 12:38–42. 10.1016/j.jacr.2014.10.002 25455196

[43] Decision Memo for Screening for Lung Cancer with Low Dose Computed Tomography (LDCT) (CAG-00439N). http://www.cms.gov/medicare-coverage-database/details/nca-decision-memo.aspx?NCAId=274&NcaName=Screening+for+Lung+Cancer+with+Low+Dose+Computed+Tomography+(LDCT)&TimeFrame=7&DocType=All&bc=AQAAIAAAAgAAAA%3d%3d& (accessed 22 Mar 2015).

[44] CDC. National Breast and Cervical Cancer Early Detection Program (NBCCEDP). http://www.cdc.gov/cancer/nbccedp/index.htm (accessed 20 Aug 2015).

[45] Lung Cancer Screening Registry—American College of Radiology. http://www.acr.org/Quality-Safety/National-Radiology-Data-Registry/Lung-Cancer-Screening-Registry (accessed 21 Aug 2015).

[46] Kauczor HU, Bonomo L, Gaga M, et al, European Society of Radiology (ESR) and European Respiratory Society (ERS). ESR/ERS White paper on lung cancer screening. Eur Radiol 2015;25:2519–31. 10.1007/s00330-015-3697-0 25929939 PMC4529446

[47] Zhao SJ, Wu N Early detection of lung cancer: low-dose computed tomography screening in China. Thorac Cancer 2015;6:385–9. 10.1111/1759-7714.12253 26273391 PMC4511314

[48] The UK NSC recommendation on Lung Cancer Screening. http://www.screening.nhs.uk/lungcancer (accessed 26 Feb 2015).

[49] ACE Programme projects | Cancer Research UK. http://www.cancerresearchuk.org/health-professional/early-diagnosis-activities/ace-programme/ace-programme-projects (accessed 25 Aug 2015).

[50] Wilson JMG, Jungner G Principles and practice of screening for disease. Geneva: World Health Organization, 1968 http://www.who.int/iris/handle/10665/37650 (accessed 25 Aug 2015).

[51] Criteria for appraising the viability, effectiveness and appropriateness of a screening programme. UK National Screening Committee https://www.gov.uk/government/publications/evidence-review-criteria-national-screening-programmes/criteria-for-appraising-the-viability-effectiveness-and-appropriateness-of-a-screening-programme (accessed 18 Sep 2015).

[52] Callister MEJ, Baldwin DR, Akram AR, et al British Thoracic Society guidelines for the investigation and management of pulmonary nodules. Thorax 2015;70 (Suppl 2):ii1–54. 10.1136/thoraxjnl-2015-207168 26082159

[53] Patz EF, Pinsky P, Gatsonis C, et al Overdiagnosis in low-dose computed tomography screening for lung cancer. JAMA Intern Med 2014;174:269–74. 10.1001/jamainternmed.2013.12738 24322569 PMC4040004

[54] Kovalchik SA, Tammemagi M, Berg CD, et al Targeting of low-dose CT screening according to the risk of lung-cancer death. N Engl J Med 2013;369:245–54. 10.1056/NEJMoa1301851 23863051 PMC3783654

[55] Field JK, Duffy SW, Baldwin DR, et al UK Lung Cancer RCT Pilot Screening Trial: baseline findings from the screening arm provide evidence for the potential implementation of lung cancer screening. Thorax 2016;71:161–70. 10.1016/S1470-2045(14)70387-0 26645413 PMC4752629

[56] Horeweg N, Th Scholten E, de Jong PA, et al Detection of lung cancer through low-dose CT screening (NELSON): a prespecified analysis of screening test performance and interval cancers. Lancet Oncol 2014;15:1342–50. 10.1016/S1470-2045(14)70387-0 25282284

[57] Slatore CG, Sullivan DR, Pappas M, et al Patient-centered outcomes among lung cancer screening recipients with computed tomography: a systematic review. J Thorac Oncol 2014;9:927–34. 10.1097/JTO.0000000000000210 24922011 PMC9208726

[58] Gareen IF, Duan F, Greco EM, et al Impact of lung cancer screening results on participant health-related quality of life and state anxiety in The National Lung Screening Trial. Cancer 2014;120:3401–9. 10.1002/cncr.28833 25065710 PMC4205265

[59] van den Bergh KAM, Essink-Bot ML, Borsboom GJJM, et al Long-term effects of lung cancer computed tomography screening on health-related quality of life: the NELSON trial. Eur Respir J 2011;38:154–61. 10.1183/09031936.00123410 21148229

[60] Bond M, Pavey T, Welch K, et al Systematic review of the psychological consequences of false-positive screening mammograms. Health Technol Assess 2013;17:1–170, v–vi 10.3310/hta17130 PMC4781086 23540978

[61] Wiener RS, Gould MK, Woloshin S, et al What do you mean, a spot?: A qualitative analysis of patients’ reactions to discussions with their physicians about pulmonary nodules. Chest 2013;143:672–7. 10.1378/chest.12-1095 22814873 PMC3590883

[62] Renzi C, Whitaker KL, Wardle J Over-reassurance and undersupport after a ‘false alarm’: a systematic review of the impact on subsequent cancer symptom attribution and help seeking. BMJ Open 2015;5:e007002 10.1136/bmjopen-2014-007002 PMC4322204 25652803

[63] Tanner NT, Gebregziabher M, Hughes Halbert C, et al Racial differences in outcomes within The National Lung Screening Trial. Implications for widespread implementation. Am J Respir Crit Care Med 2015;192:200–8. 10.1164/rccm.201502-0259OC 25928649

[64] Ruparel M, Navani N Fulfilling the Dream. Toward reducing inequalities in Lung Cancer Screening. Am J Respir Crit Care Med 2015;192:125–7. 10.1164/rccm.201505-0897ED 26177167

[65] Chiles C, Duan F, Gladish GW, et al Association of coronary artery calcification and mortality in the National Lung Screening Trial: a comparison of three scoring methods. Radiology 2015;276:82–90. 10.1148/radiol.15142062 25759972 PMC5137795

[66] Wu MT, Yang P, Huang YL, et al Coronary arterial calcification on low-dose ungated MDCT for lung cancer screening: concordance study with dedicated cardiac CT. AJR Am J Roentgenol 2008;190:923–8. 10.2214/AJR.07.2974 18356438

[67] Wille MM, Thomsen LH, Petersen J, et al Visual assessment of early emphysema and interstitial abnormalities on CT is useful in lung cancer risk analysis. Eur Radiol 2016;26:487–94. 10.1007/s00330-015-3826-9 25956938

[68] Buckens CF, van der Graaf Y, Verkooijen HM, et al Osteoporosis markers on low-dose lung cancer screening chest computed tomography scans predict all-cause mortality. Eur Radiol 2015;25:132–9. 10.1007/s00330-014-3361-0 25323601

[69] van der Aalst CM, van den Bergh KAM, Willemsen MC, et al Lung cancer screening and smoking abstinence: 2 year follow-up data from the Dutch-Belgian randomised controlled lung cancer screening trial. Thorax 2010;65:600–5. 10.1136/thx.2009.133751 20627916

[70] MacRedmond R, McVey G, Lee M, et al Screening for lung cancer using low dose CT scanning: results of 2 year follow up. Thorax 2006;61:54–6. 10.1136/thx.2004.037580 16396954 PMC2080704

[71] Schnoll RA, Miller SM, Unger M, et al Characteristics of female smokers attending a lung cancer screening program: a pilot study with implications for program development. Lung Cancer 2002;37:257–65. http://www.ncbi.nlm.nih.gov/pubmed/12234693 (accessed 26 Nov 2014). 10.1016/S0169-5002(02)00106-X 12234693

[72] Cox LS, Clark MM, Jett JR, et al Change in smoking status after spiral chest computed tomography scan screening. Cancer 2003;98:2495–501. 10.1002/cncr.11813 14635086

[73] van der Aalst CM, van Klaveren RJ, van den Bergh KAM, et al The impact of a lung cancer computed tomography screening result on smoking abstinence. Eur Respir J 2011;37:1466–73. 10.1183/09031936.00035410 21148233

[74] Ashraf H, Saghir Z, Dirksen A, et al Smoking habits in the randomised Danish Lung Cancer Screening Trial with low-dose CT: final results after a 5-year screening programme. Thorax 2014;69:574–9. 10.1136/thoraxjnl-2013-203849 24443174

[75] Anderson CM, Yip R, Henschke CI, et al Smoking cessation and relapse during a lung cancer screening program. Cancer Epidemiol Biomarkers Prev 2009;18: 3476–83. 10.1158/1055-9965.EPI-09-0176 19959698

[76] Tammemägi MC, Berg CD, Riley TL, et al Impact of lung cancer screening results on smoking cessation. J Natl Cancer Inst 2014;106:dju084 10.1093/jnci/dju084 24872540 PMC4081623

[77] Townsend CO, Clark MM, Jett JR, et al Relation between smoking cessation and receiving results from three annual spiral chest computed tomography scans for lung carcinoma screening. Cancer 2005;103:2154–62. 10.1002/cncr.21045 15825210

[78] Taylor KL, Cox LS, Zincke N, et al Lung cancer screening as a teachable moment for smoking cessation. Lung Cancer 2007;56:125–34. 10.1016/j.lungcan.2006.11.015 17196298

[79] Villanti AC, Jiang Y, Abrams DB, et al A cost-utility analysis of lung cancer screening and the additional benefits of incorporating smoking cessation interventions. PLoS ONE 2013;8:e71379 10.1371/journal.pone.0071379 23940744 PMC3737088

[80] Black WC, Gareen IF, Soneji SS, et al Cost-effectiveness of CT screening in The National Lung Screening Trial. N Engl J Med 2014;371:1793–802. 10.1056/NEJMoa1312547 25372087 PMC4335305

[81] McMahon PM, Kong CY, Bouzan C, et al Cost-effectiveness of computed tomography screening for lung cancer in the United States. J Thorac Oncol 2011;6:1841–8. 10.1097/JTO.0b013e31822e59b3 21892105 PMC3202298

[82] Doria-Rose VP, White MC, Klabunde CN, et al Use of lung cancer screening tests in the United States: results from the 2010 National Health Interview Survey. Cancer Epidemiol Biomarkers Prev 2012;21:1049–59. 10.1158/1055-9965.EPI-12-0343 22573798 PMC3392469

[83] Yankelevitz DF, Reeves AP, Kostis WJ, et al Small pulmonary nodules: volumetrically determined growth rates based on CT evaluation. Radiology 2000;217:251–6. 10.1148/radiology.217.1.r00oc33251 11012453

[84] Logan RFA, Patnick J, Nickerson C, et al Outcomes of the Bowel Cancer Screening Programme (BCSP) in England after the first 1 million tests. Gut 2012;61:1439–46. 10.1136/gutjnl-2011-300843 22156981 PMC3437782

[85] NHS Breast Screening Programme 2011 Annual Review. http://www.cancerscreening.nhs.uk/breastscreen/publications/2011review.html (accessed 19 Sep 2015).

[86] Altobelli E, Lattanzi A, Paduano R, et al Colorectal cancer prevention in Europe: burden of disease and status of screening programs. Prev Med 2014;62:132–41. 10.1016/j.ypmed.2014.02.010 24530610

[87] Altobelli E, Lattanzi A Breast cancer in European Union: An update of screening programmes as of March 2014 (review). Int J Oncol 2014;45:1785–92. http://www.spandidos-publications.com/ijo/45/5/1785/abstract (accessed 19 Sep 2015). 10.3892/ijo.2014.2632 25174328 PMC4203333

[88] van Klaveren RJ, Oudkerk M, Prokop M, et al Management of lung nodules detected by volume CT scanning. N Engl J Med 2009;361:2221–9. 10.1056/NEJMoa0906085 19955524

[89] McRonald FE, Yadegarfar G, Baldwin DR, et al The UK Lung Screen (UKLS): demographic profile of first 88,897 approaches provides recommendations for population screening. Cancer Prev Res 2014;7:362–71. 10.1158/1940-6207.CAPR-13-0206 24441672

[90] Aberle DR, Adams AM, Berg CD, et al Baseline characteristics of participants in the randomized National Lung Screening Trial. J Natl Cancer Inst 2010;102:1771–9. 10.1093/jnci/djq434 21119104 PMC2994863

[91] Hestbech MS, Siersma V, Dirksen A, et al Participation bias in a randomised trial of screening for lung cancer. Lung Cancer 2011;73:325–31. 10.1016/j.lungcan.2010.12.018 21324544

[92] Byrne MM, Davila EP, Zhao W, et al Cancer screening behaviors among smokers and non-smokers. Cancer Epidemiol 2010;34:611–17. 10.1016/j.canep.2010.06.017 20655820

[93] Fiona Dawe O Chapter 1—Smoking (General Lifestyle Survey Overview—a report on the 2011 General Lifestyle Survey). http://www.ons.gov.uk/ons/rel/ghs/general-lifestyle-survey/2011/rpt-chapter-1.html (accessed 28 Nov 2014).

[94] Siahpush M, McNeill A, Borland R, et al Socioeconomic variations in nicotine dependence, self-efficacy, and intention to quit across four countries: findings from the International Tobacco Control (ITC) Four Country Survey. Tob Control 2006;15(Suppl 3):iii71–5. 10.1136/tc.2004.008763 16754950 PMC2593052

[95] Patel D, Akporobaro A, Chinyanganya N, et al Attitudes to participation in a lung cancer screening trial: a qualitative study. Thorax 2012;67:418–25. 10.1136/thoraxjnl-2011-200055 22106018

[96] Quaife SL, McEwen A, Janes SM, et al Attitudes towards lung cancer screening within socioeconomically deprived and heavy smoking communities: a qualitative study. Lancet 2014;384:S16 10.1016/S0140-6736(14)62142-5 PMC5513004 27397651

[97] Quaife SL, McEwen A, Janes SM, et al Smoking is associated with pessimistic and avoidant beliefs about cancer: results from the International Cancer Benchmarking Partnership. Br J Cancer 2015;112:1799–804. 10.1038/bjc.2015.148 25950385 PMC4647255

[98] Ford ME, Havstad SL, Flickinger L, et al Examining the effects of false positive lung cancer screening results on subsequent lung cancer screening adherence. Cancer Epidemiol Biomarkers Prev 2003;12:28–33. http://cebp.aacrjournals.org/content/12/1/28.full (accessed 4 May 2015). 12540500

